# Delayed bowel obstruction after seat belt injury: a case report

**DOI:** 10.1186/s12876-020-01384-y

**Published:** 2020-08-08

**Authors:** Xing-Bin Ma, Bao-Guang Hu, Wei Wang, Xian-Yong Cheng, Chun-Di Guan, Cheng-Xia Liu

**Affiliations:** 1 Department of Gastroenterology and Hepatology, Binzhou Medical University Hospital, No. 661, Huanghe 2nd Road, Binzhou, 256603 Shandong China; 2 Department of Gastrointestinal Surgery, Binzhou Medical University Hospital, Shandong, China

**Keywords:** Delayed bowel obstruction, Seat belt injury, Endoscopy, Laparoscopy, Case report

## Abstract

**Background:**

Delayed bowel obstruction due to seat belt injury is extremely rare. The delayed onset of nonspecific symptoms makes a timely diagnosis difficult. A deep understanding of the characteristics of this condition is helpful for early diagnosis and treatment.

**Case presentation:**

A 39-year-old male was transferred to our hospital from another hospital complaints of progressive abdominal distension and severe weakness. In the previous hospital, he was diagnosed with “adult megacolon” and was recommended for surgical treatment. In our hospital, he was diagnosed with delayed bowel obstruction due to seat belt injury and underwent surgical intervention. Following laparoscopic adhesiolysis and resection of the narrow small intestine, his symptoms improved rapidly, and he was discharged.

**Conclusion:**

Delayed bowel obstruction due to seat belt injury may present clinical symptoms any time after the injury. Imaging examination, ileus tube and small colonoscopy may provide us with valuable cues for the diagnosis and treatment of delayed bowel obstruction, and laparoscopy may be an alternative approach in surgical intervention.

## Core tip

We reported a rare case of delayed small bowel obstruction due to seat belt injury. Based on the experience in this case, we suggest that delayed bowel obstruction due to seat belt injury may present clinical symptoms any time after the injury. Imaging examination, ileus tube and small colonoscopy may provide valuable cues for the diagnosis and treatment of delayed bowel obstruction, and laparoscopy may be an alternative approach in surgical intervention.

## Background

Bowel obstruction due to blunt abdominal trauma is common, whereas the delayed presentation of bowel obstruction following seat belt injuries is extremely rare. The delayed onset of nonspecific symptoms following seat belt injuries usually makes a timely diagnosis difficult. The underlying pathophysiological mechanism of delayed presentation following trauma remains unclear, and the characteristics of this condition have not been well described.

This report presents a rare case of delayed bowel obstruction in a male patient following seat belt injury during a car accident. Additionally, we discuss the possible mechanism for the delayed symptoms and the diagnosis and treatment of patients who experience a delayed bowel obstruction following seat belt injury.

## Case presentation

**Fig. 1:**
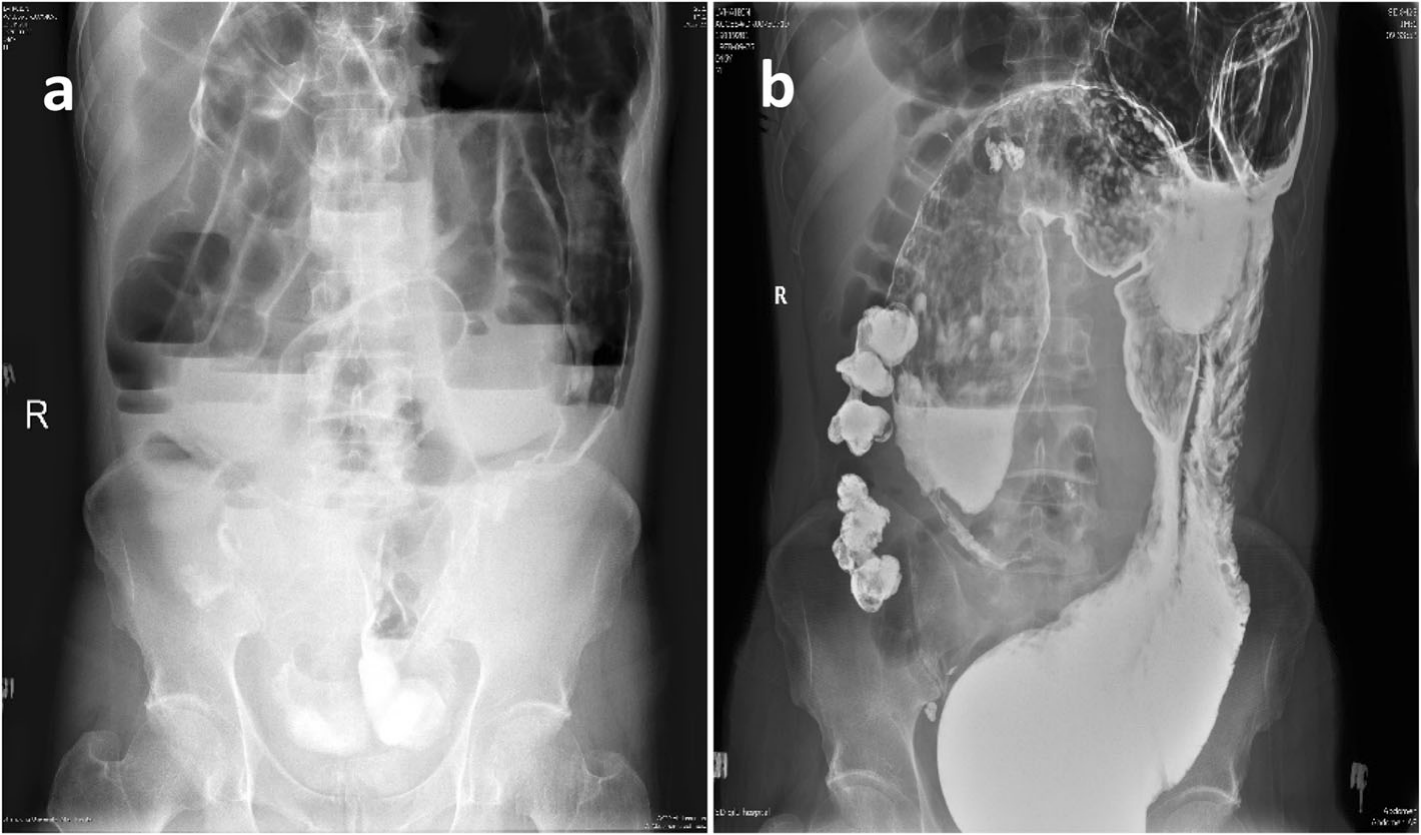
**a**-**b**. Single anteroposterior abdominal radiograph showing markedly dilated colon in the initial stage

A 39-year-old male was transferred to our hospital from another hospital. He had mild tenderness, an obvious bowel pattern and hyperactive bowel sounds; he was able to pass gas occasionally. Before admission, he suffered progressive abdominal distention and gradual deterioration, and he developed malnutrition for two months. A total alimentary tract angiography showed partial enlargement of the ascending colon and transverse colon and partial dilation of the distal small intestine (Fig. 1). He was diagnosed with “adult megacolon” and recommended for surgical treatment. However, the operation was not performed because of a significant decrease in platelets (with a minimum of 19 × 10^9^/L) and severe malnutrition. He had been in a car accident 2 years previously. He was the driver and was wearing a seat belt at the time of the accident. During that admission, he was always conscious and was found to have left clavicle fractures and multiple rib fractures. Abdominal examination showed seat belt marks and mild localized tenderness at the site of the abrasions. An abdominal CT scan showed a small amount of fluid (approximately 150 ml) in the abdominal cavity with no solid organ abnormalities. He was hemodynamically stable and was able to pass gas and defecate. He improved rapidly with conservative treatment, was discharged after several days and was asymptomatic. Two months after discharge, he started to have episodes of abdominal distension and intermittent mild tenderness, and he passed gas less frequently than before. However, he improved rapidly again after receiving treatment with traditional Chinese medicine.

**Fig. 2:**
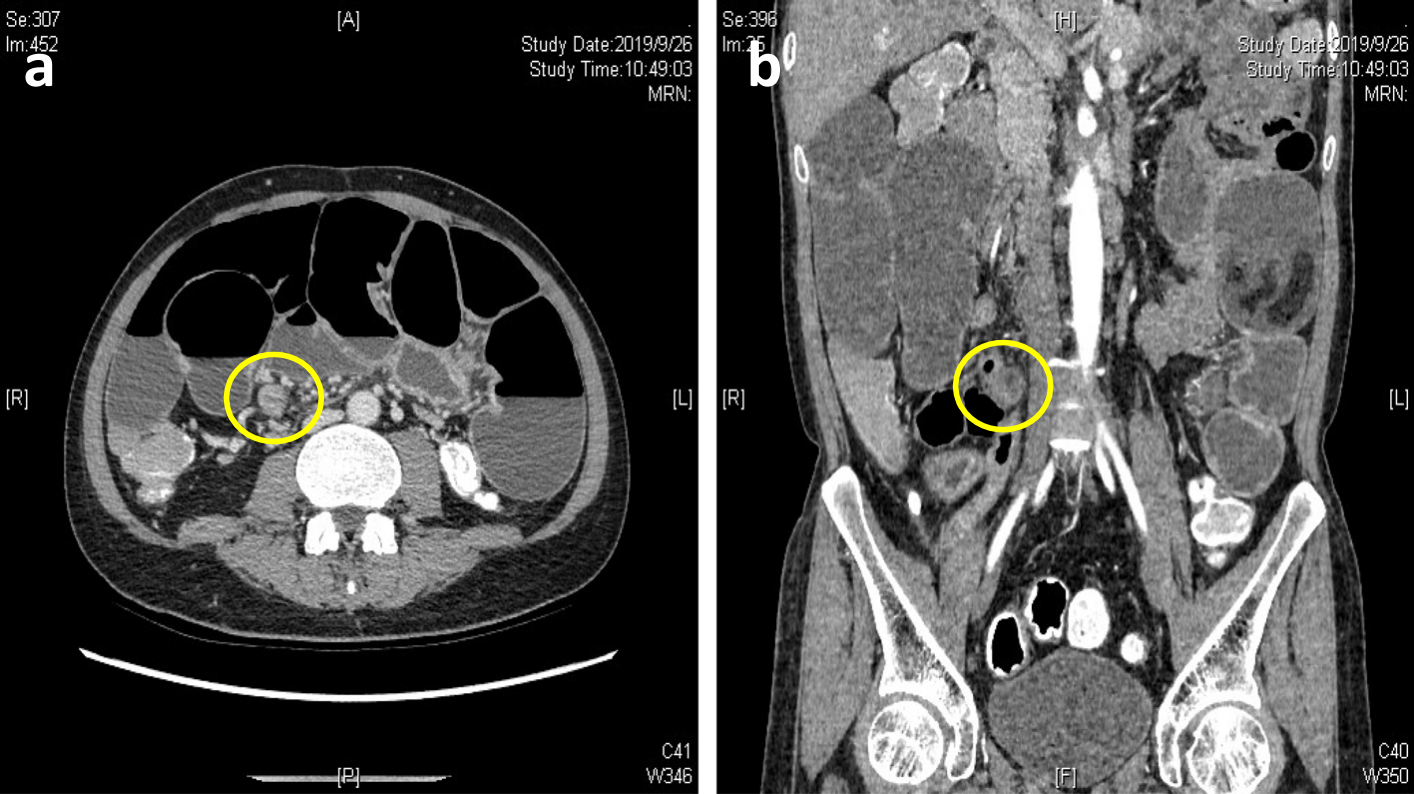
CT scan demonstrating yellow circle indicates the abrupt narrowing zone of the jejunum (**a**; cross-sectional image, **b**; coronal image)

**Fig. 3:**
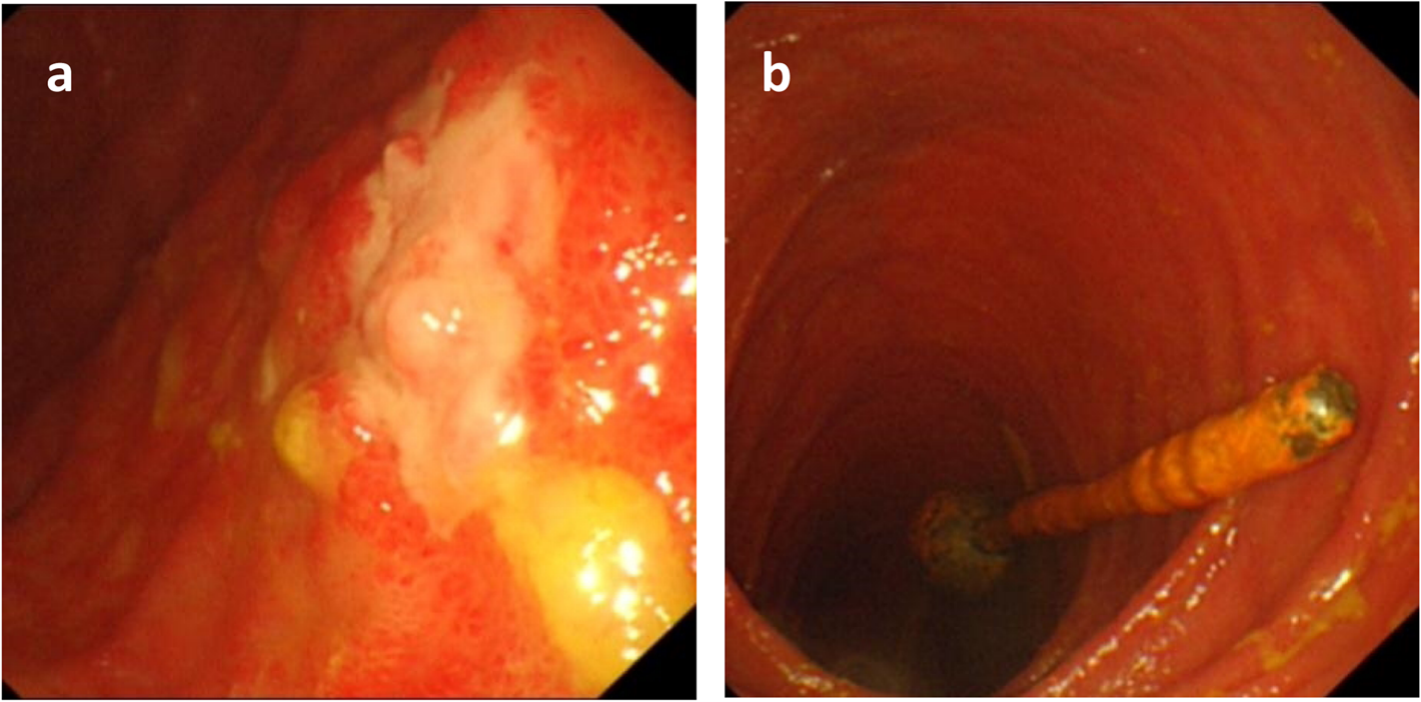
**a**. Small balloon colonoscopy can be seen mucosal congestion, edema, erosion, intestinal stenosis. **b**. The proximal dilated bowel cavity and the end of the ileus tube

**Fig. 4:**
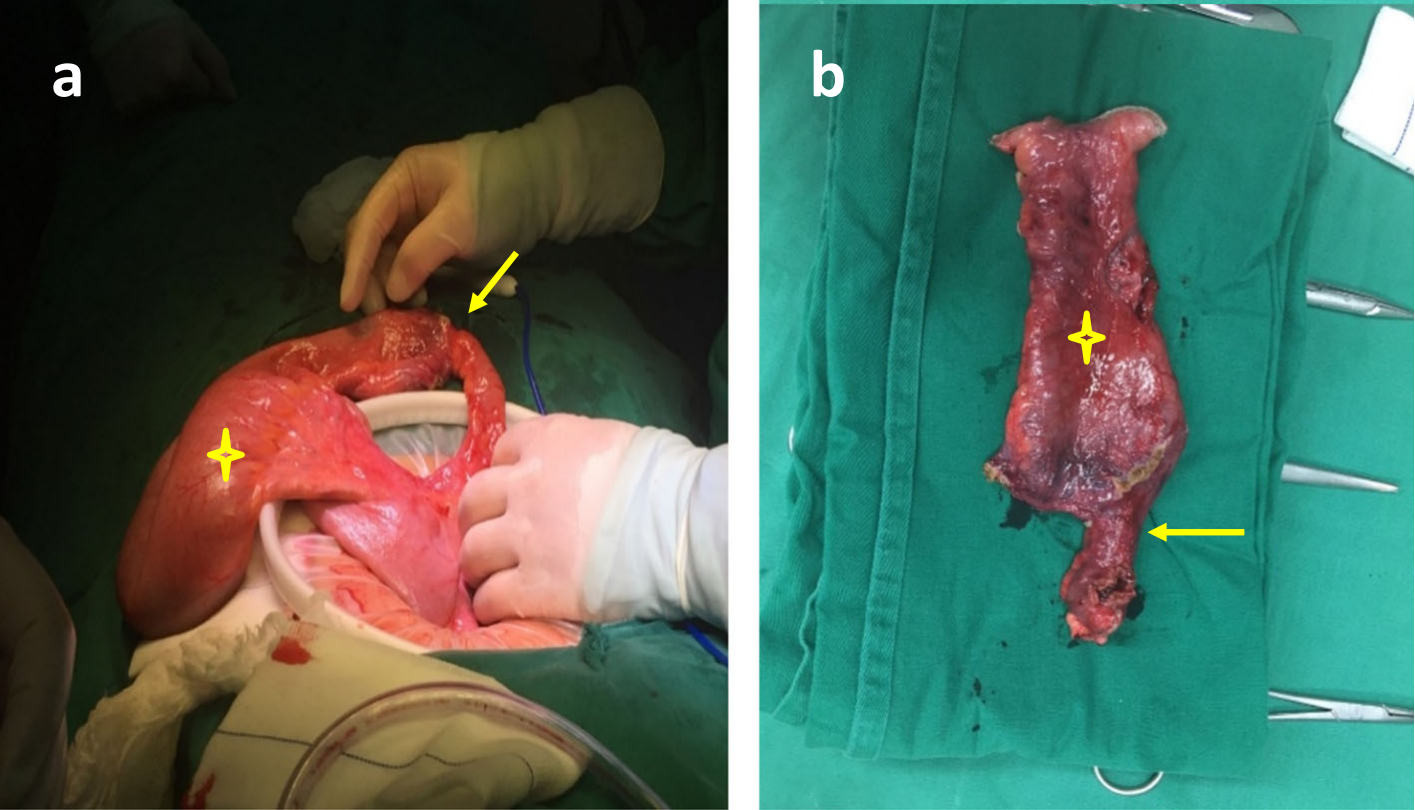
**a**-**b**. Gross image depicting the Stenosis and extremely dilated small bowel (arrow, star)

**Fig. 5:**
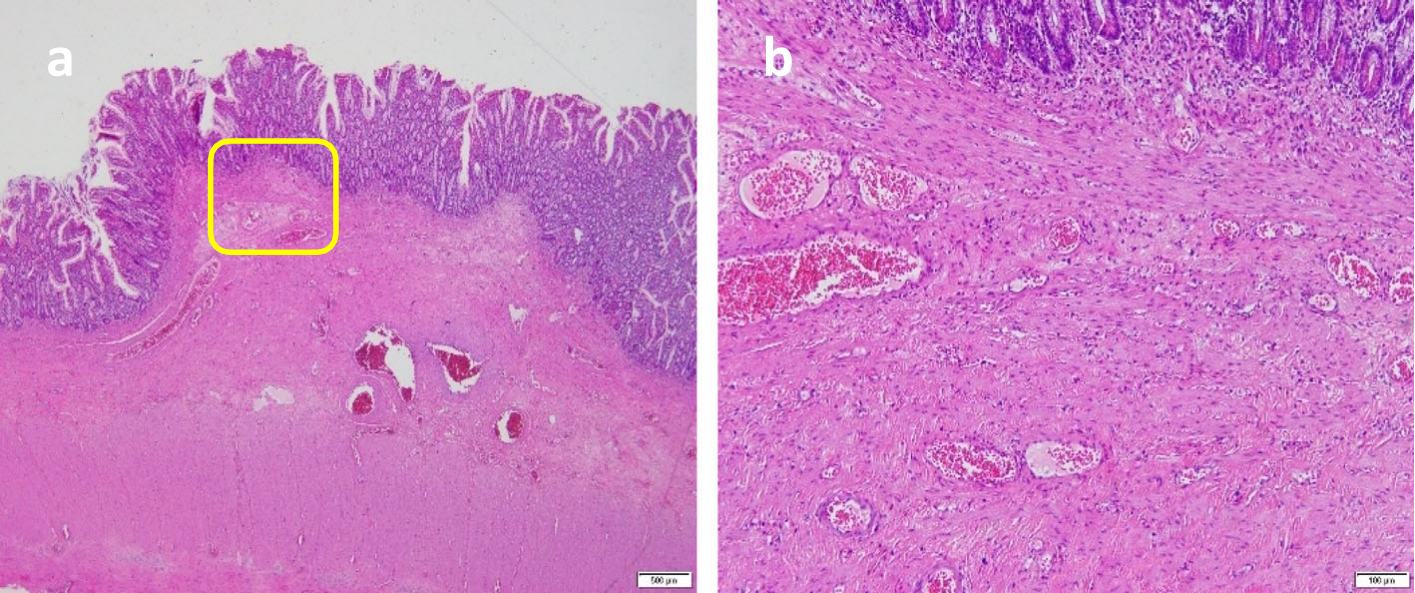
**a**-**b**. Histopathological findings. Ulcers were observed and inflammatory cells and fibroblasts infiltrated to the whole layers (**a** × 20, **b** × 100)

After admission, we first tried to improve the general condition of the patient by strengthening parenteral nutrition and correcting electrolyte imbalances. Then, a series of additional examinations were performed to explore the possible reasons for these problems. An abdominal CT scan showed an abrupt narrowing zone at the jejunum (Fig. 2). Small balloon colonoscopy found a narrow zone approximately 40–50 cm from the ileocecal valve; the surface mucosa was swollen and erosive, and the upper segment of the intestine was obviously expanded (Fig. 3). Laparoscopy was performed on the patient after multidisciplinary discussion and detailed preoperative evaluation. We found severe adhesion between the abdominal wall and intestine as well as a narrow small bowel with a length of 12 cm at approximately 40–50 cm from the ileocecal valve. The mesentery corresponding to the narrow part of the small intestine was also absent, and the proximal intestine was markedly dilatated. Additionally, a thick adhesive band was also found between the dilated proximal intestine and the sigmoid colon, and we thought it might be the main cause of colonic dilation (Fig. 4). Therefore, we performed laparoscopic adhesiolysis and partial small bowel resection, and the thick adhesive bands were destroyed. The narrow small bowel with length of 20 cm was removed. Histologically, the area was fibrotic (Fig. 5). The patient recovered rapidly and gained 5 kg in the 3 months after surgery. He was very satisfied with the treatment.

## Discussion and conclusions

The use of seat belts has significantly reduced the overall mortality associated with motor vehicle accidents. However, physicians should know that the use of seat belts is also associated with certain patterns of injury, including abdominal injuries, neck and spine injuries, chest trauma and vascular injuries [1]. Hollow viscus injury due to a seat belt is uncommon; it occurs in approximately 1% of all blunt abdominal trauma patients, and delayed small bowel obstruction (SBO) following hollow viscus injury is rarer [2, 3]. The case we reported is a typical seat belt injury, and the patient presented with a delayed SBO at 2 years after injury. The case indicates that seat belt injuries might not produce severe symptoms immediately, and the related symptoms such as bowel obstruction might present any time after injury.

Since the initial description in 1962, few articles have been published on delayed SBO due to seat belt injury [4]. Therefore, the exact cause of the obstruction remains unclear. However, the possible mechanism may be associated with a small perforation of the small intestine and perforation-induced adhesive, localized bowel ischemia, and injury to the mesentery. Most authors suggest that the most likely cause of delayed SBO due to seat belt injury is injury to the mesentery. Mesenteric injuries are commonly defined as small hematomas, contusions, or lacerations that do not compromise bowel circulation [5, 6]. Our case supports the mesenteric injury theory since there was a large mesenteric defect corresponding to the narrow part of the small intestine. In addition, we believe that delayed bowel obstruction was also due to a combination of posttraumatic ischemia and the adhesive between the small intestine and sigmoid colon.

Preoperative diagnosis for patients with delayed bowel obstruction due to seat belt injuries remains a challenge to surgeons. A patient may be relatively asymptomatic, have stable vital signs, have no clinical evidence for peritonitis, and may even have negative initial image [7] . In most cases, CT findings are often subtle and nonspecific. The presence of free intraperitoneal fluid in the abdomen without any evidence of solid organ injury may be the sole piece of evidence of a significant bowel injury at the first CT evaluation on first admission [8]. Moreover, multislice computed tomography enterography may help to identify the location of the obstruction when delayed bowel obstruction occurs. Additionally, small colonoscopy may be helpful in the differential diagnosis of delayed bowel obstruction; this procedure not only helps to identify the location of the obstruction but also helps to investigate the cause or nature of the lesion. In the current case, the presence of free intraperitoneal fluid at the first CT evaluation, the narrow small intestine observed in the secondary imaging examination, and the findings from the small colonoscopy provided us with valuable cues for the preoperative diagnosis of delayed bowel obstruction due to seat belt injury. Meanwhile, the application of ileus tubes also greatly aided in bowel preparation, diagnosis and treatment in the current case.

However, there is still debate regarding the optimum duration of conservative management and the timing of surgery for SBO, especially when the SBO is due to seat belt injuries, because no high-quality studies have been performed to examine these issues [9, 10]. To date, most data with beneficial effects are from case reports or observational studies that enrolled a limited number of patients. The presence of free intraperitoneal fluid in the abdomen is not observed in stable patients [11]. Open surgery has been the preferred method for surgical treatment of strangulating adhesive SBO and SBO that is refractory to conservative management. Currently, laparoscopic approaches have become increasingly popular because of their multiple advantages such as being minimally invasive and having potentially better outcomes than traditional approaches. Laparoscopic approaches can also be applied for SBO and trauma to assess and treat intra-abdominal adhesions and abdominal injuries [12, 13]. In this case, we first performed a laparoscopy and laparoscopic adhesiolysis. Then, we removed the narrow small intestine and reconstructed the digestive tract via a small abdominal incision. The patient recovered rapidly after surgical intervention. Our case revealed that laparoscopy might be useful in delayed bowel obstruction due to seat belt injury. However, we must note that laparoscopic adhesiolysis is not feasible for all patients or all surgeons, and a detailed preoperative evaluation is essential.

Based on our experience and knowledge of the reported cases in the literature, we propose that delayed bowel obstruction due to seat belt injury may present clinical symptoms any time after the injury, and these patients should be closely monitored. Imaging examination, ileus tube and small colonoscopy may provide valuable cues for the diagnosis and treatment of delayed bowel obstruction, and laparoscopy may be an alternative approach for surgical intervention.

## Data Availability

Data sharing is not applicable to this article as no datasets were generated or analyzed during the current study.

## Abbreviations

SBO: small bowel obstruction
CT: Computed tomography
Kg: kilogram
